# Tuning NaYF_4_ Nanoparticles through Alkaline Earth Doping

**DOI:** 10.3390/nano3040583

**Published:** 2013-10-24

**Authors:** Xian Chen, Dengfeng Peng, Feng Wang

**Affiliations:** 1 Department of Physics and Materials Science, City University of Hong Kong, 83 Tat Chee Avenue, Hong Kong; E-Mails: zjuchenxian@gmail.com (X.C.); dengpeng@cityu.edu.hk (D.P.); 2 City University of Hong Kong Shenzhen Research Institute, Shenzhen 518057, China

**Keywords:** impurity doping, nanoparticles, phase transformation, upconversion

## Abstract

Phase and size of lanthanide-doped nanoparticles are the most important characteristics that dictate optical properties of these nanoparticles and affect their technological applications. Herein, we present a systematic study to examine the effect of alkaline earth doping on the formation of NaYF_4_ upconversion nanoparticles. We show that alkaline earth doping has a dual function of tuning particle size of hexagonal phase NaYF_4_ nanoparticles and stabilizing cubic phase NaYF_4_ nanoparticles depending on composition and concentration of the dopant ions. The study described here represents a facile and general strategy to tuning the properties of NaYF_4_ upconversion nanoparticles.

## 1. Introduction

The synthesis of lanthanide-doped upconversion (UC) nanoparticles with well-defined phase and size is important for understanding the enormously complex optical transitions in the host lattice [1,2,3] and for realizing applications of these nanoparticles in solid-state lasers, three-dimensional flat-panel display, solar cells and especially biolabeling and bioimaging [4,5,6,7,8,9,10,11,12,13,14,15]. The phase and size of lanthanide-doped nanoparticles are usually tuned by controlling the thermodynamic or kinetic growth of the nanoparticles through manipulation of several experimental variables, such as temperature, reaction time, and relative concentrations of the precursors [16,17,18,19,20]. The major problems of these approaches includes the difficulty in fine tune the nanoparticle size over a broad range and the need for extreme reaction conditions such as toxic reactants and high temperatures [21,22,23].

Impurity doping that involves incorporating suitable foreign ions into a host lattice has recently emerged as a promising alternative to control the formation of lanthanide-doped nanoparticles [24,25,26,27,28,29]. The doping approach usually allows the fine tuning of a variety of nanoparticle properties including phase, size, and even shape following a facile and standard synthetic procedure by simple modification of dopant composition and concentration [24,25,26,27,28,29,30,31,32,33,34]. Doping also modifies free energies of a materials system and stabilizes a particular crystal phase or particle morphology that is typically inaccessible by a common synthetic method [35,36].

For better understanding and control of doping-mediated crystal growth process, herein we describe the synthesis and characterization of a series of NaYF_4_:Yb/Er nanoparticles in the presence of alkaline earth dopant ions of varying composition (Sr^2+^ and Ca^2+^) and concentration (0–60 mol %). As one of the most studied upconversion materials, NaYF_4_ nanoparticles generally crystallize in either the cubic or hexagonal form. We show that the Sr^2+^ or Ca^2+^ dopant ions can significantly modify the phase and size of the as-synthesized NaYF_4_:Yb/Er nanoparticles through precise control of dopant concentration. We also correlate upconversion emission of the nanoparticles with the dopant composition and concentration by optical measurement.

## 2. Results and Discussion

**Figure 1:**
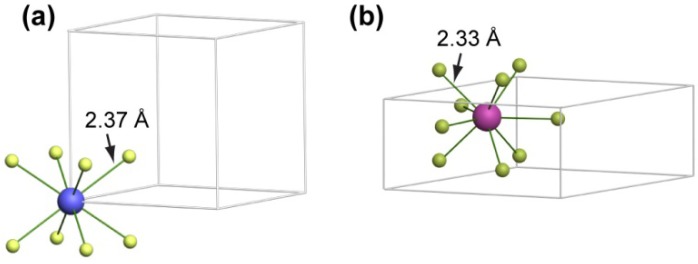
A comparison of the crystal site for the metal ions in the (**a**) cubic and (**b**) hexagonal phase NaYF_4_.

Previous investigations suggest that in wet chemical synthesis NaYF_4_ nanoparticles usually crystallize in cubic phase at first, which spontaneous transformed to the corresponding hexagonal phase as the reaction proceeds [16,37]. The cubic to hexagonal phase transformation of NaYF_4_ is accompanied by a distortion and compression in electron cloud of the cation ions to accommodate the more asymmetric and dense hexagonal crystal structure (Figure 1). We reasoned that the presence of impurities of significant larger ionic size than Y^3+^ (*r* = 1.159 Å) [38] in the host lattice should suppress the phase transformation by increasing the energy barrier, thereby stabilizing the cubic phase product.

**Figure 2:**
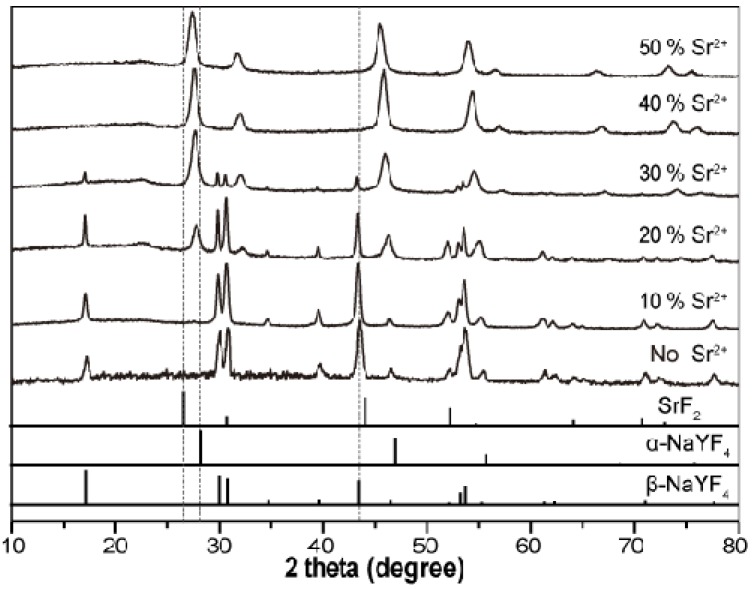
X-ray diffraction (XRD) patterns of the NaYF_4_:Yb/Er (18/2 mol %) nanoparticles obtained in the presence of different amount of Sr^2+^ dopant ions. The bottom line spectra are literature data for cubic phase SrF_2_ (JCPDS file number 06-0262 [40]), cubic phase NaYF_4_ (JCPDS file number 77-2042 [41]) and hexagonal phase NaYF_4_ (JCPDS file number 16-0334 [39]), respectively.

As a proof-of-concept experiment, Sr^2+^ ions (*r* = 1.40 Å) [38] that are optically inactive and chemically compatible with trivalent lanthanide ions were introduced to modify the growth process of NaYF_4_:Yb/Er nanoparticles. A series of samples obtained in the presence of Sr^2+^ ions at different concentrations (0–50 mol %) were first examined by X-ray diffraction (XRD). As shown in Figure 2, XRD pattern of the Sr^2+^ free sample can be indexed as pure hexagonal phase NaYF_4_ (JCPDS file number 16-0334 [39]), which is consistent with previous studies [16,24]. On doping with increased Sr^2+^ concentrations, the preservation of the cubic phase in these samples is evident and pure cubic phase NaYF_4_:Yb/Er nanoparticles was obtained when the Sr^2+^ ion concentration reached 40 mol %. The observed single cubic phase at a significantly high Sr^2+^ dopant concentration (50 mol %) can be attributed to the small structural difference between the cubic phase NaYF_4_ and SrF_2_. Notably, the diffraction peaks shift towards low diffraction angles with increasing dopant concentration of Sr^2+^, which results from expansion in unit-cell volume due to the larger Sr^2+^ dopant ions and confirms the formation of a homogeneous Y-Sr solid solution.

**Figure 3:**
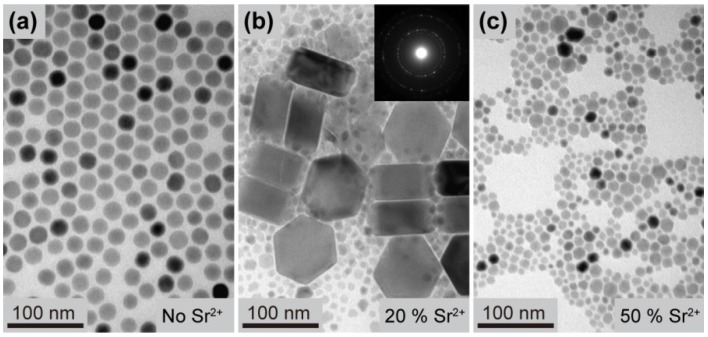
Transmission electron microscopy (TEM) images of the NaYF_4_:Yb/Er (18/2 mol %) nanoparticles obtained in the presence of (**a**) 0 mol %, (**b**) 20 mol %, and (**c**) 50 mol % of Sr^2+^ dopant ions, respectively.

Transmission electron microscopy (TEM) characterizations were then carried out to study the morphology and size of NaYF_4_:Yb/Er (18/2 mol%) samples doped with Sr^2+^ ions at different concentrations. Figure 3a presents the Transmission electron microscopy (TEM) image of the NaYF_4_:Yb/Er nanoparticles without Sr^2+^ doping and shows uniform nanoparticles with a spherical morphology. In the presence of Sr^2+^ (20 mol %), the TEM images shows two distinct particle morphologies including small irregular particles and large hexagon prisms (Figure 3b), which is consistent with the presence of two phases observed by XRD. Selected area electron diffraction analysis reveals that the small nanoparticles are cubic phase NaYF_4_ (Figure 3b, inset). We therefore concluded the large prisms to be hexagonal phase NaYF_4_. The increase in particle size of the hexagonal phase product is attributed to the increased positive charge density associated with substitutional Sr^2+^ ions at the nanoparticle surface, which promote the nanoparticle growth and results in a larger particle size [42]. With increasing dopant concentration of Sr^2+^ (50 mol %), the large prisms disappeared (Figure 3c), suggesting the formation of pure cubic phase NaYF_4_ nanoparticles.

**Figure 4:**
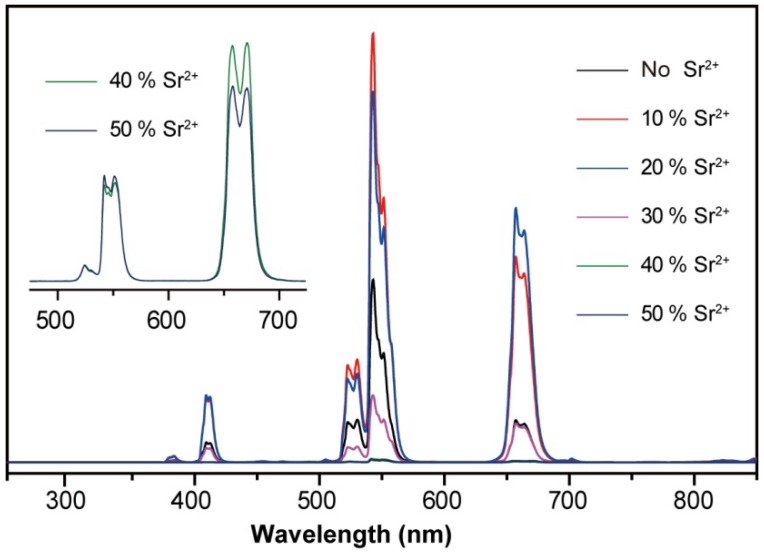
Upconversion emission spectra of the NaYF_4_:Yb/Er (18/2 mol %) nanoparticles obtained in the presence of different amount of Sr^2+^ dopant ions. Inset: amplified spectra of the nanoparticles comprising high dopant concentration of Sr^2+^.

We next investigated optical properties of the NaYF_4_:Yb/Er nanoparticles as a function of Sr^2+^ dopant concentration. Figure 4 depicts the upconversion emission spectra of the nanoparticles doped with varying amount of Sr^2+^ ion under 976-nm diode laser excitation. Characteristic emission peaks at 409 nm (blue), 526 and 545 nm (green), and 660 nm (red) were observed and assigned to ^2^H_9/2_→^4^I_15/2_, ^2^H_11/2_, ^4^S_3/2_→^4^I_15/2_, and ^4^F_9/2_→^4^I_15/2_ transitions of Er^3+^, respectively. The spectra shows that the emission intensity is slightly enhanced at low Sr^2+^ content (<20 mol %), which is attributed to the formation of large hexagonal prism. Further increase in Sr^2+^ dopant concentration results in a sharp decrease in the emission intensity because of the phase transformation from hexagonal to cubic. The amplified spectra (inset) reveal that the red-to-green intensity ratio is largely increased at high Sr^2+^ content, which is partly due to the formation of small sized cubic phase nanoparticles.

**Figure 5:**
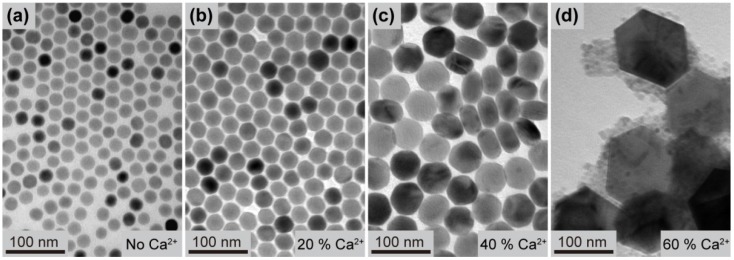
TEM images of the NaYF_4_:Yb/Er (18/2 mol %) nanoparticles obtained in the presence of (**a**) 0 mol %, (**b**) 20 mol %, (**c**) 40 mol %, and (**d**) 60 mol % of Ca^2+^ dopant ions, respectively.

In a further set of experiments, we examined the effect Ca^2+^ doping on crystal growth process of the NaYF_4_:Yb/Er nanoparticles. TEM images (Figure 5) of the as-synthesized nanoparticles all show no phase separation for dopant concentrations below 40 mol %, suggesting that the cubic phase was not effectively stabilized by Ca^2+^ ions. The observation can be ascribed to the smaller ionic size of Ca^2+^ ions (*r* = 1.26 Å) [38] than that of Sr^2+^ and therefore the relatively high amenability to the phase transformation into the dense hexagonal structure. Nevertheless, the nanoparticle size is fine-tuned through precise control of the Ca^2+^ dopant concentration. The steady increase in the nanoparticle size with increasing Ca^2+^ dopant concentration is mainly due to surface charge modification similar as for the Sr-doped samples.

## 3. Experimental Section

Y(CH_3_COOH)_3_·*x*H_2_O (99.9%), Yb(CH_3_COOH)_3_·*x*H_2_O (99.9%), Er(CH_3_COOH)_3_·*x*H_2_O (99.9%), Sr(CH_3_COOH)_2_·2H_2_O, Ca(CH_3_COOH)_2_ (≥99%), NaOH (>98%), NH_4_F (>98%), 1-octadecene (90%), oleic acid (90%), were all purchased from Sigma-Aldrich and used as starting materials without further purification.

In a typical procedure for the synthesis of NaYF_4_:Yb/Er nanoparticles, 2 mL aqueous solution (0.2 M) of *RE*(CH_3_COOH)_3_ (*RE* = Y, Yb and Er) and *AE*(CH_3_COOH)_2_ (*AE* = Sr and Ca) was added to a 50 mL flask containing oleic acid (3 mL) and 1-octadecene (7 mL). The mixture was heated at 110 °C for 30 min to remove water and subsequently at 150 °C for 1 h before cooling to 50 °C. Then 5 mL methanol solution containing 1.6 mmol NH_4_F and 1 mmol NaOH was added and the solution was stirred at 50 °C for 60 min. The solution was then heated at 110 °C under vacuum for 10 min to remove methanol and moisture, followed by heating at 300 °C for 60 min under argon atmosphere. After cooling down to room temperature, excessive amount of ethanol was poured into the solution. The resultant mixture was separated by centrifugation, washed with cyclohexane and ethanol several times, and finally redispersed in cyclohexane.

The crystal structure of the samples was analyzed by X-ray diffraction analysis (XRD, Rigaku SmartLab, Tokyo, Japan) using a Cu Kα radiation. Transmission electron microscopy (TEM) measurements were carried out on a Philips CM-20 transmission electron microscope (FEI, Hillsboro, OR, USA) operating at an acceleration voltage of 200 kV. Photoluminescence spectra were recorded at room temperature with an F-4600 spectrophotometer (Hitachi, Tokyo, Japan) with the excitation source adapted to a fiber couple diode laser. All spectra were obtained at room temperature from cyclohexane dispersions of the nanoparticles (1 wt.%) at an excitation power density of 20 W cm^−^^2^.

## 4. Conclusions

In summary, we have demonstrated an alkaline earth doping approach to tuning the size and phase of NaYF_4_ nanoparticles following a consistent synthetic procedure. Sr^2+^ dopant ions are found to promote the formation of cubic phase NaYF_4_, mainly owing to the large ionic size that resists dense atom packing in the hexagonal structure. By contrast, Ca^2+^ dopant ions with a relatively small ionic size only display weak effect on phase transformation of the NaYF_4_ nanoparticle. However, it allows fine-tuning of the nanoparticle size through surface charge modification. The doping approach described here represents a general strategy to expand the range of nanoparticle products that can be accessed by a particular synthetic method.
